# Downregulation of p300/CBP‐associated factor inhibits cardiomyocyte apoptosis via suppression of NF‐κB pathway in ischaemia/reperfusion injury rats

**DOI:** 10.1111/jcmm.16959

**Published:** 2021-10-03

**Authors:** Liqiang Qiu, Xiaoxiong Liu, Wenjing Li, Zhebo Liu, Changwu Xu, Hao Xia

**Affiliations:** ^1^ Department of Cardiology Renmin Hospital of Wuhan University Wuhan China; ^2^ Cardiovascular Research Institute Wuhan University Wuhan China; ^3^ Hubei Key Laboratory of Cardiology Wuhan China; ^4^ Department of Integrated Traditional Chinese and Western Medicine Tianyou Hospital Affiliated to Wuhan University of Science and Technology Wuhan China; ^5^ Department of Cardiology The Central Hospital of Wuhan Tongji Medical College Huazhong University of Science and Technology Wuhan China

**Keywords:** apoptosis, hypoxia, inflammation, myocardial ischaemia‐reperfusion injury, P300/CBP‐associated factor

## Abstract

Cardiomyocyte apoptosis is the main reason of cardiac injury after myocardial ischaemia‐reperfusion (I/R) injury (MIRI), but the role of p300/CBP‐associated factor (PCAF) on myocardial apoptosis in MIRI is unknown. The aim of this study was to investigate the main mechanism of PCAF modulating cardiomyocyte apoptosis in MIRI. The MIRI model was constructed by ligation of the rat left anterior descending coronary vessel for 30 min and reperfusion for 24 h in vivo. H9c2 cells were harvested after induced by hypoxia for 6 h and then reoxygenation for 24 h (H/R) in vitro. The RNA interference PCAF expression adenovirus was transfected into rat myocardium and H9c2 cells. The area of myocardial infarction, cardiac function, myocardial injury marker levels, apoptosis, inflammation and oxidative stress were detected respectively. Both I/R and H/R remarkably upregulated the expression of PCAF, and downregulation of PCAF significantly attenuated myocardial apoptosis, inflammation and oxidative stress caused by I/R and H/R. In addition, downregulation of PCAF inhibited the activation of NF‐κB signalling pathway in cardiomyocytes undergoing H/R. Pretreatment of lipopolysaccharide, a NF‐κB pathway activator, could blunt these protective effects of PCAF downregulation on myocardial apoptosis in MIRI. These results highlight that downregulation of PCAF could reduce cardiomyocyte apoptosis by inhibiting the NF‐κB pathway, thereby providing protection for MIRI. Therefore, PCAF might be a promising target for protecting against cardiac dysfunction induced by MIRI.

## 1 INTRODUCTION

Ischaemic heart disease is seriously endangering human health and causing severe public health threats.^1^, ^2^ Ischaemic heart disease will become the second leading cause of death in rural and urban areas worldwide in 2030 according to the data from WHO.^2^ Clinical studies have found that early restoration of coronary blood flow is particularly important to reduce myocardial necrosis and save life for ischaemic heart disease patients, but rapid reperfusion itself could also cause myocardial ischaemia‐reperfusion injury (MIRI), which may be the main cause for a poor prognosis.^3^ Although the mechanism of MIRI is complex, more and more studies have shown that cardiomyocyte apoptosis, and the levels of reactive oxidative stress (ROS) and inflammation upregulation play a crucial role in the progression of MIRI.^4^, ^5^ Therefore, prevention and suppression of these pathological processes have become a consensus for the treatment of MIRI.

P300/CBP‐associated factor (PCAF) is a transcriptional coactivator with acetyltransferase activity, and is involved in regulating various cellular activities and signalling pathways.^6^ Previous studies showed that PCAF is involved in regulating apoptosis, oxidative stress and inflammation. PCAF promotes apoptosis of liver cancer cells by inhibiting Serine/Threonine protein kinase 1,^7^ and hydrogen peroxide have been proved to stimulate the expression of PCAF during oxidative stress.^8^ In addition, downregulation of PCAF could obviously inhibit the production of pro‐inflammatory factors in angiogenesis and restenosis.^6^, ^9^ It's also found that garcinol, a natural inhibitor of PCAF, could reduce inflammation, promote apoptosis and possess the effect of anti‐oxidation.^10^, ^11^ Moreover, our previous study found that downregulation of PCAF could reduce myocardial damage caused by I/R, which mainly due to the inhibition of excessive autophagic activity of cardiomyocytes.^12^ However, it is unknown whether PCAF could attenuate MIRI by inhibiting cardiomyocyte apoptosis.

In this study, we hypothesized that downregulation of PCAF ameliorated cardiac impairment and myocardial damage via reducing cardiomyocyte apoptosis, oxidative stress and inflammation after I/R injury. We used a rat MIRI model for in vivo experiments and hypoxia‐reoxygenation (H/R) model with H9c2 cells for in vitro experiments to figure out the roles and underlying mechanisms of PCAF on cardiomyocyte apoptosis in MIRI.

## 2 MATERIALS AND METHODS

### 2.1 Materials and agents

Cell Bank of the Chinese Academy of Sciences provided the H9c2 cell line (#GNR5). All the cell growth medium used in this study was obtained from HyClone (#SH30023.01B). Foetal bovine serum (FBS) was bought from Tianhang Biotechnology Company (#11011‐8611). Lipopolysaccharide (LPS) was purchased from Beyotime Biotechnology (#S1732), Cell counting assay Kit 8 (CCK‐8) was obtained from Dojindo (#CK04). The commercial ELISA assay kits were purchased from Elabscience Biotechnology and used to detected the levels of the inflammatory cytokine such as IL‐6, TNF‐α. The levels of oxidative stress were evaluated by Superoxide dismutase (SOD, #A001‐3), Catalase (CAT, #A007‐1–1) Lactate dehydrogenase (LDH), Creatine kinase (CK), CK isoenzymes (CK‐MB), and Malondialdehyde (MDA) assay commercial kits, all of above kits were obtained from Jiancheng Bioengineering Institute. Cardiac troponin I (cTnT) assay kit from Roche Diagnostics GmbH. Flow cytometry apoptosis kit was purchased from BD Biosciences (#550474); TUNEL assay kit purchased from Roche (#11684817910). Dihydroethidium (DHE) was from Beyotime Biotechnology (#S0063). The primary antibodies of GAPDH, Bax and Bcl‐2 were purchased from Sigma‐Aldrich. The primary antibodies of PCAF, α‐actinin, NF‐κB p65and phosphorylation of NF‐κB p65 Ser536 were purchased from Cell Signaling Technology.

### 2.2 The construction of adenoviral vectors

The adenovirus with siRNA against the rat PCAF (Ad‐PCAF‐RNAi) was produced by GeneChem. The sense sequence of siRNA for rat PCAF gene is 5′‐GACAAACTGCCTCTTGAGAAA‐3′, and 5′‐TTCTCCGAACGTGTCACGT‐3′ for the control. The virus was amplified, purified and routinely titrated to 2 × 10^10^ PFU/ml.

### 2.3 In situ heart adenoviral transfection

The animal care procedure adhered to the Principles of Laboratory Animal Care, and the protocol of animal experiments were reviewed and approved by the Animal Care and Use Committee of Renmin Hospital of Wuhan University. All of Sprague‐Dawley rats (200–250 g) were randomly allotted into four groups: Sham surgery group (Sham group), Ischaemia‐reperfusion group (I/R group), left ventricle transfected with Ad‐GFP 48 h ahead of I/R (I/R + Ad‐GFP group), and left ventricle transfected with Ad‐PCAF RNAi 48 h ahead of I/R (I/R + Ad‐PCAF RNAi group), and *n* = 10 for each group. The experiment procedure in detail was described previously.^13^

### 2.4 Cell culture and hypoxia‐reoxygenation

H9c2 cells were maintained in a cell incubator at 37℃ and supplemented with 5% CO_2_. The Ad‐GFP or Ad‐PCAF‐RNAi virus was transfected into H9c2 cells in serum‐free medium at a multiplicity of infection (MOI) of 30. After the virus was successfully transfected, the H/R model was established subsequently. Briefly, the H9c2 cells were cultured in hypoxia, glucose and serum‐free DMEM buffer for 6 h. After that, the cells were cultured with DMEM/F12 medium containing 10% FBS and exposed to 5% CO_2_ and 95% air for 24 h.

### 2.5 Triphenyltetrazolium chloride (TTC) staining

The Triphenyltetrazolium chloride (TTC) staining was used to determinate the infarct size of heart immediately after I/R. After 24 h reperfusion, all the samples were sliced into 1 mm thickness. Then the slices of heart were placed in 2% TTC solution at 37℃ for 30 min and photographed digitally. The total area of left ventricle and size of infarction and were measured by Image J software, and the rate of infarct of the left ventricle was calculated by the percentage of the infarct size to the area of left ventricular.

### 2.6 Evaluation of rat heart function

The parameters of left ventricular fraction shortening (LVFS) and left ventricular ejection fraction (LVEF) were detected by Doppler echocardiography and used to evaluate the systolic function after I/R.

### 2.7 Detection of injury and inflammatory markers of cardiomyocytes

Commercial assay kits were used to detect the content of LDH, CK, CK‐MB, cTnT, TNF‐α and IL‐6 in blood samples and cell supernatants. All procedures were carried out according to the kits' instructions. The activity of enzyme was expressed as international units per litre.

### 2.8 CCK‐8 assay

The CCK‐8 assay was conducted to evaluate the cell viability, and the experimental process were performed as previously described.^14^ Briefly, the cells were cultured in a 96‐well plate at 2.0 × 10^4^ cells/well. When the cells reached 60% confluence, the corresponding intervention was applied. After the intervention was completed, 10 μl of CCK‐8 reagent was added to each well, and the cells were incubated for 2 h. Finally, the optical density (OD) value of each well was measured on a microplate reader (450 nm).

### 2.9 TUNEL staining

Myocardial apoptosis was detected with TUNEL staining assay following the manufacturer's instruction.^15^

### 2.10 Flow cytometry

For apoptosis detection, 2 × 10^5^ cells were washed with 500 μl binding buffer, subsequently incubated with 5 μl PI and 5 μl Annexin V‐APC for 15 min at room temperature in dark. The apoptotic cells were detected by flow cytometry.

### 2.11 Evaluation of ROS production

The ROS level of myocardial tissue and H9c2 cells were detected by dihydroethidium (DHE). Specifically, the frozen sections of rat heart were incubated with 10 μM DHE at 37°C for 30 min in dark. Then the image was captured with a fluorescence microscope. For the detection of ROS in H9c2 cells, the cells were washed three times with PBS, and then cultured in serum‐free medium containing 5 μM DHE for 30 min at 37℃ in dark. The intensity of fluorescence was quantified by Image J software.

### 2.12 Detection of SOD, CAT and MDA

The contents of SOD, CAT and MDA were detected with assay kits in accordance with the manufacturer's instructions.

### 2.13 Western blot

Western blot was conducted as our previously described.^16^ The primary antibodies of Bax (1:1000), Bcl‐2 (1:1000), NF‐κB p65 (1:1000), phospho‐NF‐κB p65 Ser536 (1:1000), PCAF (1:1000) and GAPDH (1:2000) were used respectively.

### 2.14 Immunofluorescence analysis

The fixed cell slides were permeabilized for 20 min at room temperature, and then washed with PBS and blocked with goat serum for 30 min. After that, the blocking solution was removed with absorbent paper. Next, the primary antibody diluent was added and incubated overnight at 4℃. The next day, the slides were washed, and diluted fluorescent secondary antibody was added and the slides were incubated in a humidified box for 1 h. DAPI was added to stain the nuclei. Finally, the slides were mounted with an anti‐fluorescence quencher, and images were collected under a fluorescence microscope.

### 2.15 Co‐immunoprecipitation

The cells were collected and centrifuged at 875 g  for 3 min, the supernatant was discarded, and the precipitate was resuspended in 500 μl of 150 mmol/L immunoprecipitation (IP) buffer to lyse the cells. Then, the lysate was placed on ice for 30 min and mixed several times at intervals of 10 min. Next, the lysate was centrifuged at 14008 g for 10 min at 4°C, and 50 μl of the supernatant was taken as the input, and the remaining supernatant was used as the IP sample. Then 10 μl of beads was added to the lysed protein sample to remove impurities. New beads were added to the protein sample, and the corresponding antibody was added and incubated at 4°C for 3 h. Afterwards, the sample was centrifuged at 1191 g for 2 min, the supernatant was discarded, and the beads were washed twice with IP buffer for 5 min each time. After the beads were washed, loading buffer was added, and the sample was heated at 95°C for 10 min to complete the preparation of the protein sample. Then, the input and IP samples were electrophoresed individually, and Western blotting was used to measure the expression of related proteins.

### 2.16 Statistical analysis

All the data were expressed as mean ± SD. SPSS 22.0 software was used for statistical analysis. Analysis of variance was used for multiple comparisons and least significant difference *t* test for post‐hoc tests. *p* < 0.05 was considered to be statistically significant.

## 3 RESULTS

### 3.1 I/R increased PCAF expression and downregulation of PCAF attenuates MIRI in vivo

**FIGURE 1:**
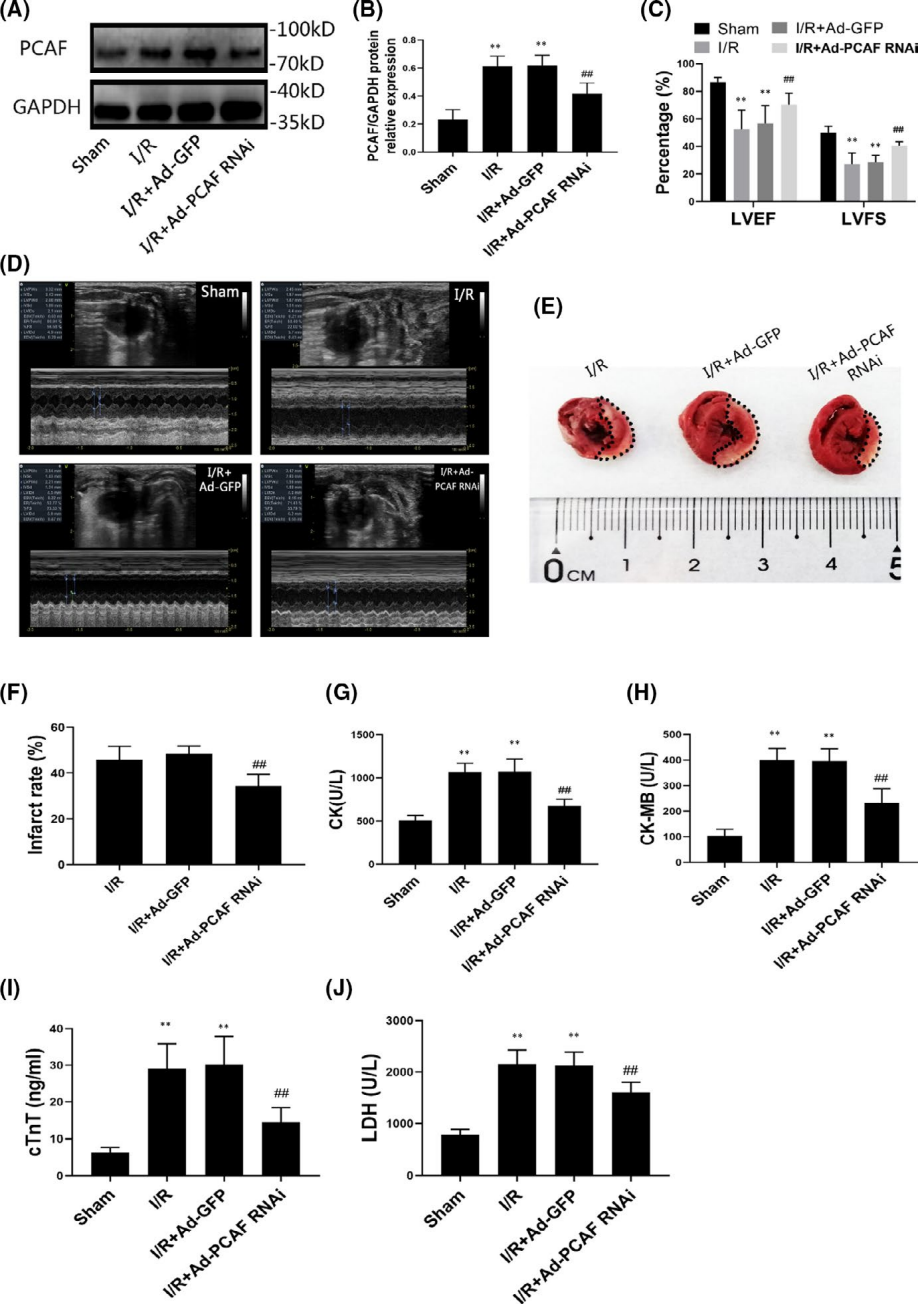
I/R increased the expression of p300/CBP‐associated factor (PCAF) and downregulation of PCAF attenuates MIRI in vivo. (A‐B) I/R increased the expression of PCAF in protein levels (*n* = 5). (C‐D) The quantifications of left ventricular ejection fraction (LVEF) and left ventricular fraction shortening (LVFS) were performed with echocardiography (*n* = 5). (E‐F) TTC staining was used to measure the area of myocardial infarction (*n* = 5). (G‐J) The activity of creatine kinase (CK), CK isoenzymes (CK‐MB), cardiac troponin I (cTnT) and lactate dehydrogenase (LDH) in the different groups (*n* = 5). **p < 0.01 compare with the Sham group; ^##^ *p* < 0.01 compare with I/R + Ad‐GFP group or I/R + Ad‐PCAF RNAi group

To explore the role of PCAF in MIRI, the PCAF expression was detected in vivo. As presented in Figure 1A, the expression of PCAF in I/R group was remarkably increased compared to the Sham group in protein levels (*p* < 0.05). In order to verify the downregulation effect of adenoviral transfection, the protein level of PCAF in the rat heart was also measured and the expression of PCAF significantly decreased after Ad‐PCAF RNAi transfection (Figure 1B).

I/R exposure significantly impaired the cardiac function, which was manifested as a decrease in LVEF and LVFS compared to the Sham group (both *p* < 0.05, Figure 1C,D). However, Doppler ultrasound study showed that LVEF and LVFS were significantly increased after PCAF downregulation (LVEF: 70.17 ± 7.92% in I/R + Ad‐PCAF RNAi group vs. 56.48 ± 12.34% in I/R + Ad‐GFP group; LVFS: 40.52 ± 2.93% vs. 28.81 ± 4.75, both *p* < 0.05). In addition, compared with the I/R + Ad‐GFP group, pretreatment with Ad‐PCAF RNAi significantly decreased the infarct rate of the left ventricle (*p* < 0.05, Figure 1E,F). Moreover, downregulation of PCAF decreased the serum content of CK, CK‐MB, cTnT and LDH (all *p* < 0.05, Figure 1G‐J). These results suggested that downregulation of PCAF played a cardioprotective role in MIRI.

### 3.2 PCAF downregulation alleviated I/R‐induced apoptosis

**FIGURE 2:**
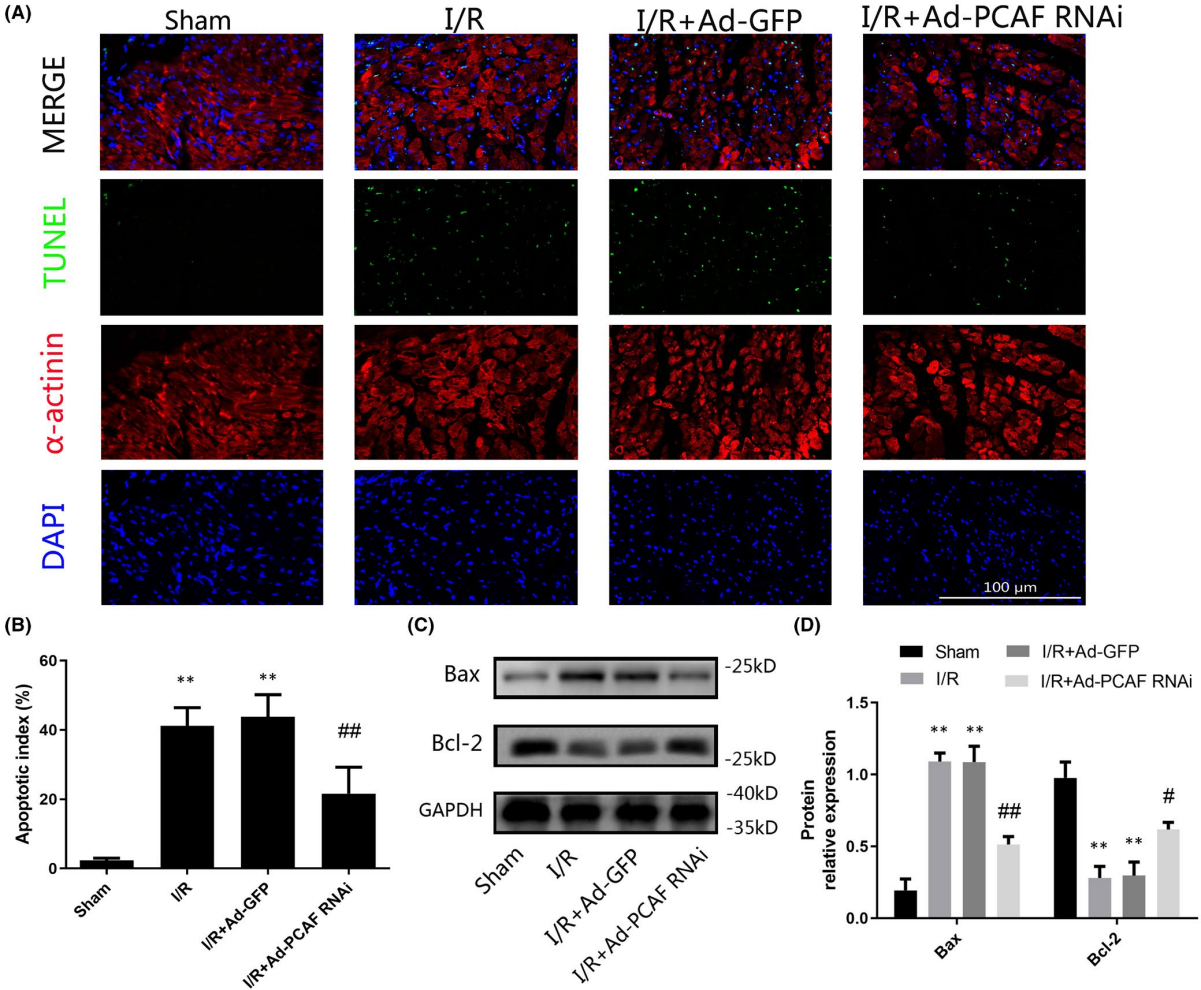
Downregulation of p300/CBP‐associated factor (PCAF) alleviated I/R‐induced apoptosis. (A) Representative immunofluorescences of TUNEL (green), α‐actinin (red), and DAPI (blue) staining in the infarct border zone. The scale bar is 100 µm, 400×. (B) Quantitative analysis of TUNEL‐positive cardiomyocytes in the infarct border zone at 24 h after I/R (*n* = 5). (C‐D) The expression levels of Bax and Bcl‐2 (*n* = 6). **p < 0.01 compare with the Sham group; ^#^ *p* < 0.05, ^##^ *p* < 0.01 compare with the I/R + Ad‐GFP group

To figure out the potential involvement of apoptosis in the cardioprotective role of PCAF downregulation in MIRI, we performed TUNEL staining and Western blot assays. Our results suggested that the rate of TUNEL‐positive cells in I/R group and I/R + Ad‐GFP group were remarkably higher than that in the Sham group (Figure 2A). However, PCAF downregulation markedly reduced the rate of TUNEL‐positive myocytes compared with that in I/R + Ad‐GFP group (27.94 ± 2.43% vs. 42.78 ± 2.87%, *p* < 0.05; Figure 2B). Meanwhile, downregulation of PCAF significantly decreased the expression level of pro‐apoptotic Bax protein in cardiomyocytes and increased anti‐apoptotic Bcl‐2 protein levels relative to those in I/R + Ad‐GFP group (both *p* < 0.05, Figure 2C,D).

### 3.3 Downregulation of PCAF reduces I/R‐induced inflammation and oxidative stress

**FIGURE 3:**
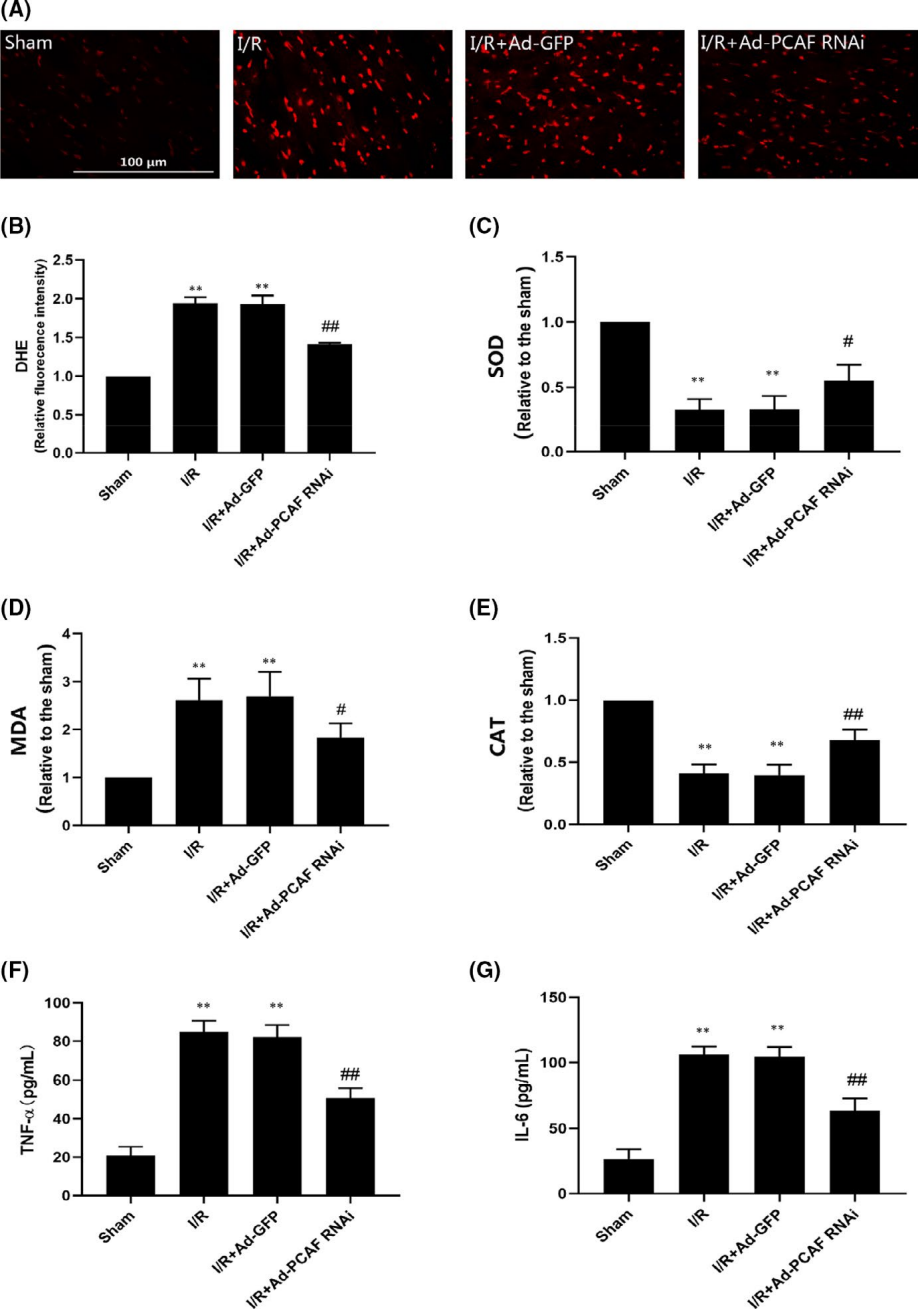
Downregulation of p300/CBP‐associated factor (PCAF) inhibits I/R‐induced inflammation and oxidative stress. (A‐B) The reactive oxidative stress (ROS) level was detected by dihydroethidium (DHE) immunofluorescence probe in different groups. The scale bar is 100 µm, 400×. (C‐E) superoxide dismutase (SOD) activity, malondialdehyde (MDA) and catalase (CAT) contents were detected by commercial kits. The levels of TNF‐α and IL‐6 were analysed by ELISA assays (F‐G). *n* = 5 for each group. **p < 0.01 compare with Sham group; ^#^ *p* < 0.05, ^##^ *p* < 0.01 Compare with I/R + Ad‐GFP group

We detected the levels of oxidative stress and inflammation in rat myocardial tissue and serum. As shown in Figures 3, I/R potently increased the levels of ROS and MDA and decreased the enzyme activities of CAT and SOD in the I/R group and I/R + Ad‐GFP group (both *p* < 0.05). However, downregulation of PCAF could partially reversed the above‐mentioned changes (both *p* < 0.05, Figure 3A‐E). Meanwhile, I/R increased the contents of IL‐6 and TNF‐α in serum of rat, while downregulation of PCAF could effectively reduce the serum levels of TNF‐α and IL‐6 in MIRI rats (both *p* < 0.05, Figure 3F‐G).

### 3.4 Downregulation of PCAF alleviates H/R injury and apoptosis in H9c2 cells

**FIGURE 4:**
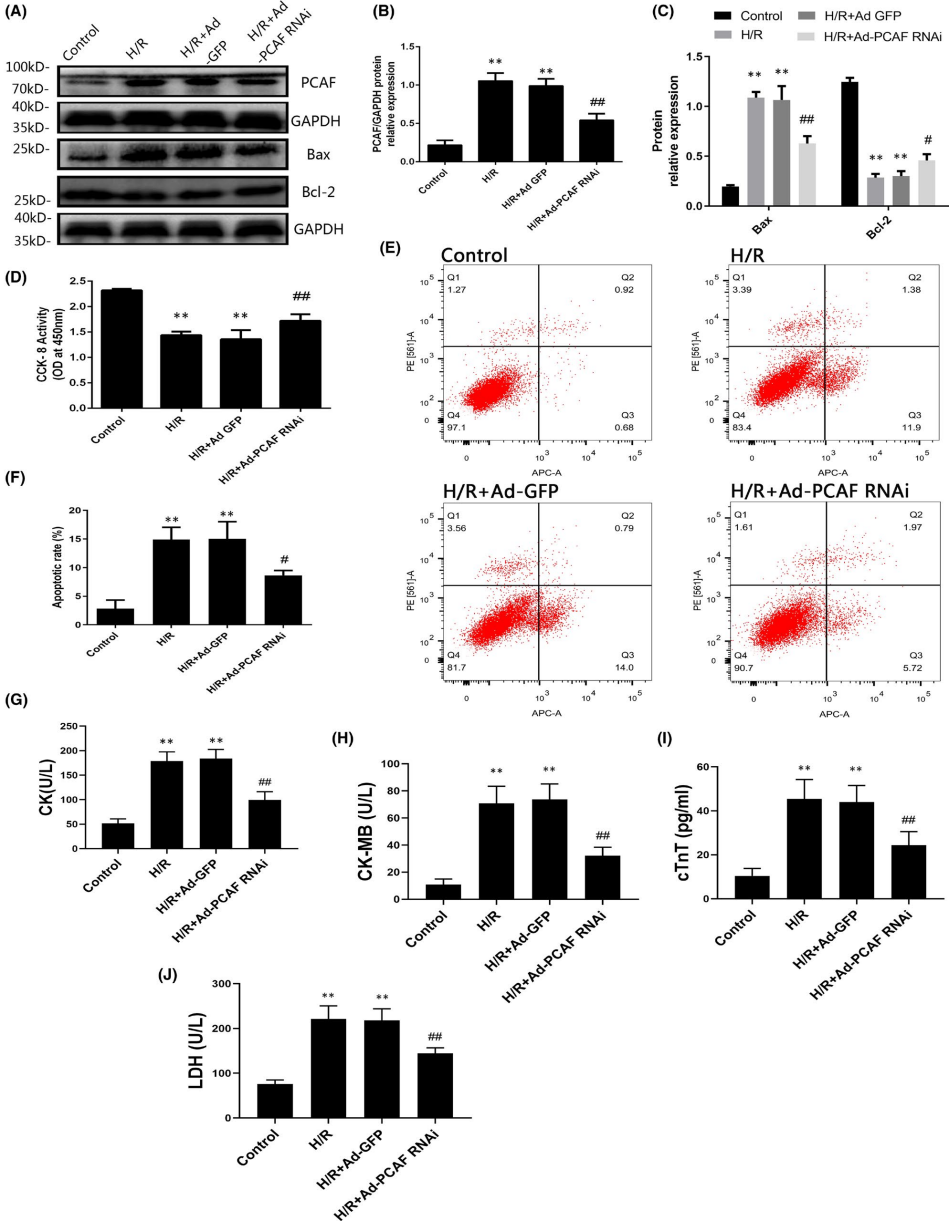
Decreasing the expression of p300/CBP‐associated factor (PCAF) alleviated apoptosis during H/R in H9c2 cells. (A‐C) The expression of PCAF, Bax and Bcl‐2 in each group (*n* = 6). (D) H9c2 cells viability were analysed by CCK‐8 assay (*n* = 6). (E‐F) The apoptosis rate of H9c2 induced by H/R in different group were detected via flow cytometry assay (*n* = 3). (G‐J) The content of creatine kinase (CK), CK isoenzymes (CK‐MB), cardiac troponin I (cTnT) and lactate dehydrogenase (LDH) in cell culture medium were detected by commercial kits (*n* = 5). ^**^ *p* < 0.01 compare with Control group; *^#^p* < 0.05, ^##^ *p* < 0.01 compare with the H/R + Ad‐GFP group

The H9c2 cells were exposed to hypoxia for 6 h and then reoxygenated for 24 h. H/R significantly promoted the expression of PCAF in H9c2 cells, and the transfection of Ad‐PCAF RNAi obviously reduced the expression of PCAF (both *p* < 0.05, Figure 4A,B). Expectedly, in the H/R and H/R + Ad‐GFP groups, Bcl‐2 was significantly downregulated, and the pro‐apoptotic protein Bax was upregulated markedly. However, these changes were suppressed after PCAF downregulation (*p* < 0.05, Figure 4A,C).

To evaluate the protective effect of PCAF downregulation on H9c2 cardiomyocytes after H/R, CCK‐8 assay was used to assess the cell viability. As shown in Figure 4D, the cell viability significantly decreased in the H/R and H/R + Ad‐GFP groups, while PCAF downregulation markedly increased the cell viability. Meanwhile, the apoptosis rate was significantly upregulated in H/R group and H/R + Ad‐GFP group compared with that in the Control group, while downregulation of PCAF markedly alleviated cell apoptosis induced by H/R (*p* < 0.05, Figure 4E,F). Moreover, the content of CK, CK‐MB, cTnT and LDH were significantly increased in H/R and H/R + Ad‐GFP group, and were partially reversed by downregulation of PCAF in H9c2 cells (all *p* < 0.05, Figure 4G‐J).

### 3.5 Downregulation of PCAF inhibits H/R‐induced oxidative stress and inflammation in H9c2 cells

**FIGURE 5:**
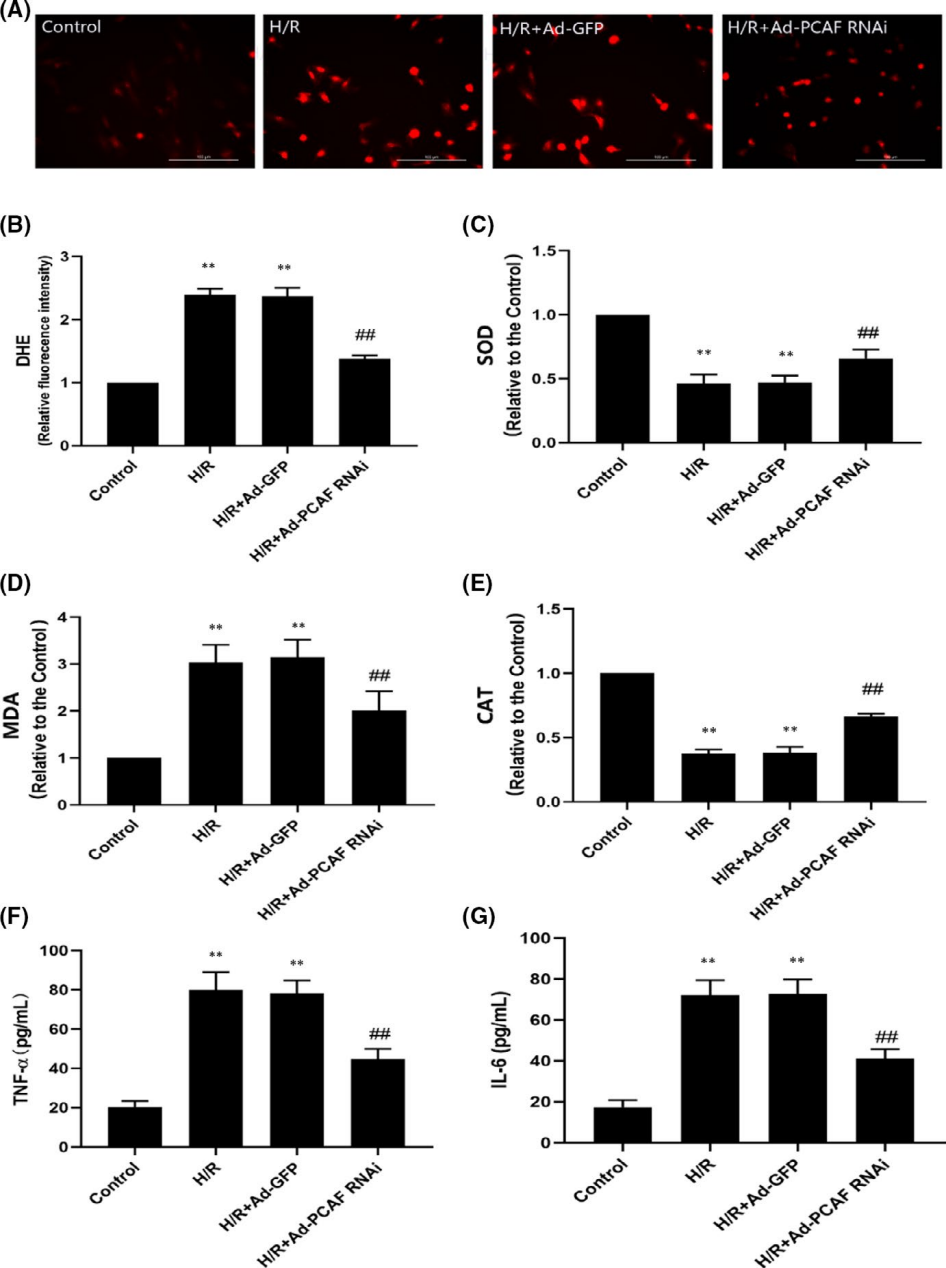
Downregulation of p300/CBP‐associated factor (PCAF) inhibits H/R‐induced inflammation and oxidative stress in H9c2 cells. (A‐B) The representative images of intracellular reactive oxidative stress (ROS) formation were detected by dihydroethidium (DHE) staining. The scale bar is 100 µm, 200×. (C‐E) The enzymatic activities of superoxide dismutase (SOD), catalase (CAT) and malondialdehyde (MDA) were detected by commercial kits. (F‐G) The content of TNF‐α and IL‐6 were detected by ELISA kits. *n* = 5 per group. **p < 0.01 compare with the Control group; ^##^ *p* < 0.01 compare with H/R + Ad‐GFP group

As presented in Figure 5A,B, the ROS activity was increased in H/R group compared to that in the Control group (*p* < 0.05). However, the levels and activity of ROS was significantly decreased in H/R + Ad‐PCAF RNAi group (*p* < 0.05). In addition, downregulation of PCAF significantly inhibited MDA levels and rescued SOD and CAT activities (all *p* < 0.05, Figure 5C‐E). Moreover, the levels of TNF‐α and IL‐6 were significantly increased in the H/R group, while downregulation of PCAF attenuated these changes (both *p* < 0.05, Figure 5F‐G).

### 3.6 The protective effect of PCAF downregulation in MIRI depends on the inactivation of the NF‐κB pathway

**FIGURE 6:**
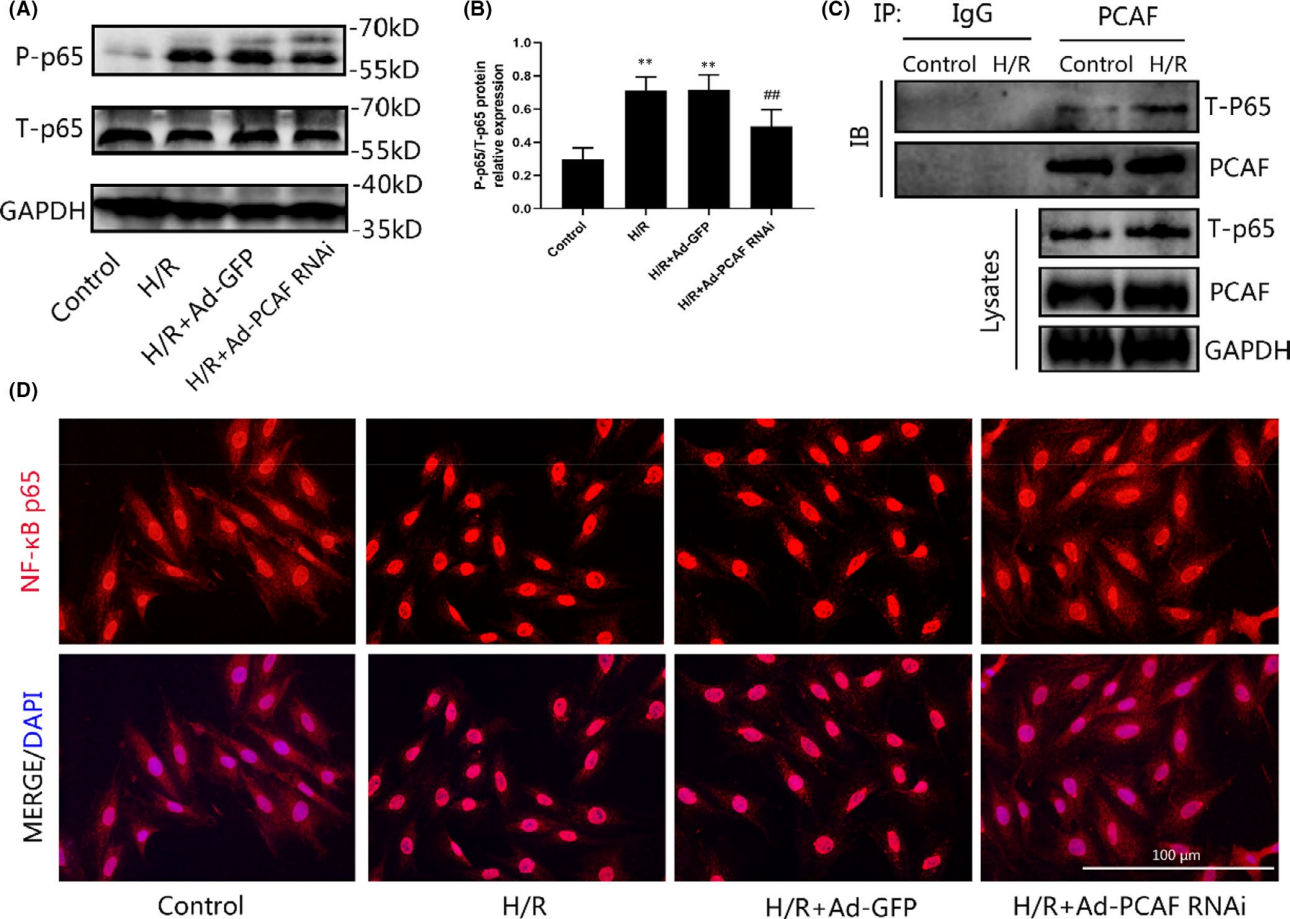
p300/CBP‐associated factor (PCAF) downregulation inactivates the NF‐κB pathway in H/R injury. (A‐B) The expression of phosphorylation of NF‐κB p65 and total NF‐κB p65 (*n* = 5). (C) Co‐immunoprecipitation was used to detect the interaction between PCAF and NF‐κB p65 (*n* = 3). (D)The immunofluorescence detects the nuclear translocation of NF‐κB p65. The scale bar is 100 µm, 400×. *n* = 5 per group. **p < 0.01 vs. Control group; ^##^ *p* < 0.01 vs. H/R + Ad‐GFP group

NF‐κB was a recognized transcription factor in modulation of inflammation, oxidative stress and cell apoptosis in the context of MIRI.^17^, ^18^, ^19^ The expression level of phospho‐NF‐κB p65 was significantly upregulated after H/R compared with the control cells. While H9c2 cells were transfected with Ad‐shRNA‐PCAF, the protein level of phospho‐NF‐κB p65 was markedly decreased (*p* < 0.05, Figure 6A,B). The results of Co‐IP showed that there was a direct interaction between PCAF and NF‐κB p65, and after H/R induction, the interaction between PCAF and NF‐κB became stronger (Figure 6C). For further analysis of the impact of PCAF downregulation on NF‐κB pathway, we used immunofluorescence to explore the nuclear translocation of NF‐κB p65. As expected, H/R promoted the transfer of NF‐κB p65 into the nucleus in H9c2 cells, while nuclear translocation of NF‐κB p65 was blunted in cells pretreated with Ad‐PCAF RNAi (Figure 6D).

**FIGURE 7:**
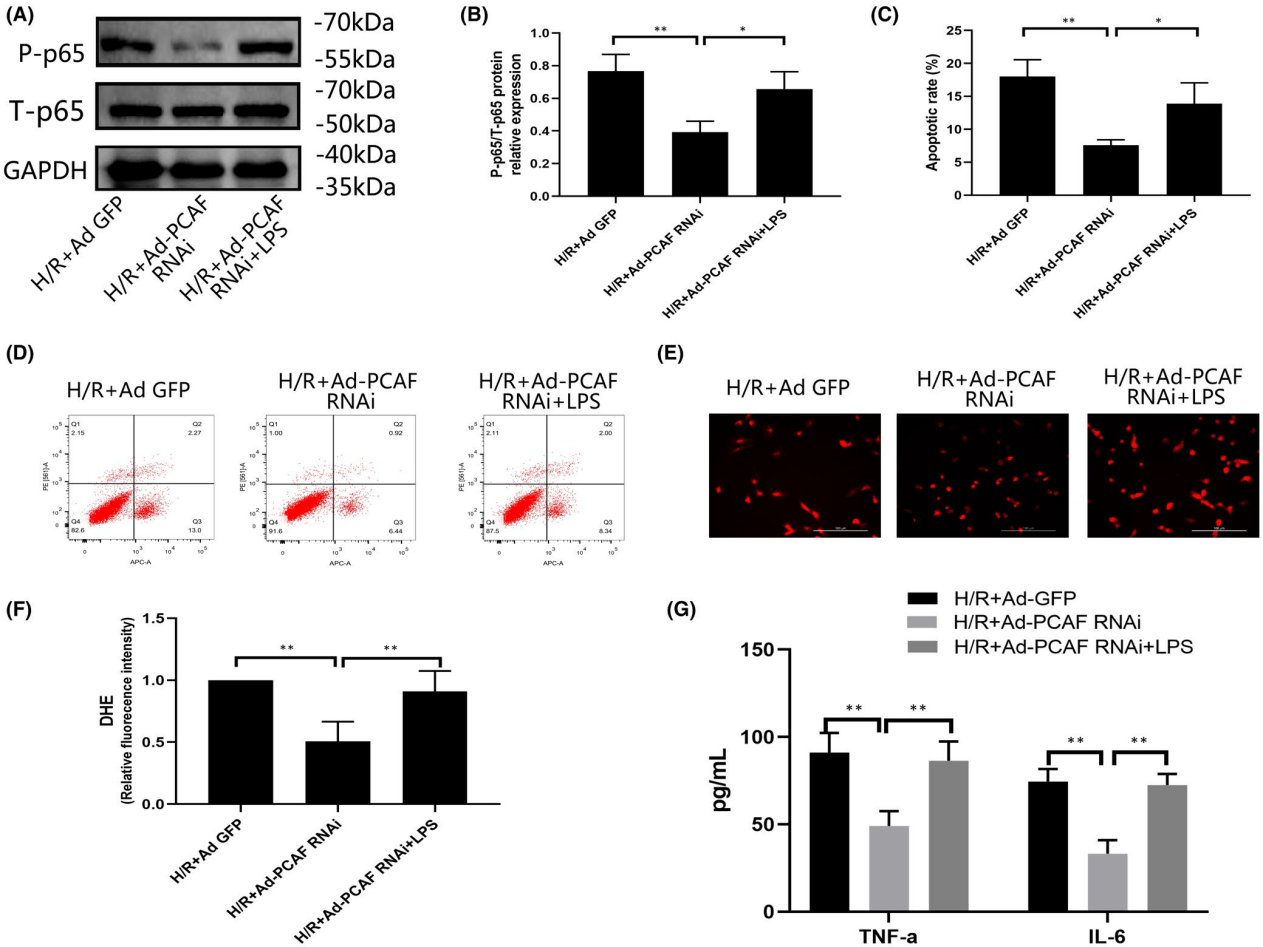
Activation of NF‐κB pathway blunted the beneficial effects of p300/CBP‐associated factor (PCAF) downregulation against H/R injury. (A‐B) The NF‐κB p65 and Phosphorylation NF‐κB p65 protein levels in each group (*n* = 3). (C‐D) Detected the apoptotic rate in each group by flow cytometry (*n* = 5). **p < 0.01; *p < 0.05. (E‐F) Dihydroethidium (DHE) immunofluorescence probe were used to evaluate intracellular reactive oxidative stress (ROS) formation (*n* = 5). The scale bar is 100 µm, 200×. (G) The level of TNF‐α and IL‐6 was measured by ELISA kits (*n* = 5). **p < 0.01

Based on the above results, we subsequently conducted a rescue experiment. After transfected with adenovirus, H9c2 cells were treated with LPS, a potent NF‐κB activator, and then carried out H/R treatment. Compared with H/R + Ad‐PCAF RNAi group, the expression of phospho‐NF‐κB p65 was significantly increased in H/R + Ad‐PCAF RNAi + LPS group (*p* < 0.05, Figure 7A,B). Meanwhile, LPS treatment stimulated the apoptosis of H9c2 cells (*p* < 0.05, Figure 7C,D). In addition, LPS treatment remarkably enhanced the fluorescence intensity of DHE (*p* < 0.05, Figure 7E,F). Moreover, compared with H/R + Ad‐PCAF RNAi group, the content of TNF‐α and IL‐6 in cells were all increased in H/R + Ad‐PCAF RNAi + LPS group (both *p* < 0.05, Figure 7G).

## 4 DISCUSSION

This study found I/R increased the expression of PCAF both in vivo and in vitro, and downregulation of PCAF significantly improved the heart function and reduced myocardial infarction area and markers of myocardial injury. Meanwhile, downregulation of PCAF significantly inhibited cardiomyocyte apoptosis, oxidative stress and inflammation. In addition, the protective effect of PCAF downregulation in MIRI may depends on the inhibition of the NF‐κB pathway. Therefore, all these findings revealed that the cardioprotective effects of PCAF downregulation against MIRI was mediated via inhibiting NF‐κB pathway and then suppression of inflammation, ROS and apoptosis.

As a member of Gcn5‐related N‐acetyltransferase family, PCAF is dominantly expressed in the complexes with acetyltransferase activity. A series of previous studies found that PCAF plays a crucial role in cardiovascular diseases. Our previous work found that PCAF knockdown significantly restrained neointimal formation in rat carotid artery balloon injury model.^6^ Moreover, the knockdown of PCAF alleviated myocardial reperfusion injury by inhibition of cardiomyocyte autophagy.^12^ It is well known that apoptosis, oxidative stress and inflammation are vital driver in progression of pathological process of MIRI. Recent studies demonstrated that PCAF may involve in the progress of cell apoptosis, oxidative stress, inflammation and metabolism.^20^ Our findings in this study indicated that MIRI was accompanied by an increased expression of PCAF, both in vivo and in vitro. Importantly, downregulation of PCAF significantly reduced myocardial damage and improved cardiac function. At the same time, levels of oxidative stress, inflammation and apoptosis were also significantly inhibited. These results further confirmed that downregulation of PCAF was beneficial for alleviating MIRI.

Previous studies have shown that the process of myocardial ischaemia and reperfusion could produce many free radicals, destroy the body's oxidation and antioxidant processes, lead to lipid peroxidation, and cause irreversible damage to capillaries and tissue cells.^21^ MDA is the end product of lipid peroxidation, which causes biological macromolecules to polymerize with each other, and is cytotoxic. Middle or high concentrations of ROS could induce inflammatory response and even apoptosis in cardiomyocytes through oxidative stress.^17^, ^22^ SOD is the main enzyme that eliminates ROS in cells. It can restore the dynamic balance of oxidation and anti‐oxidation, and prevent ROS from causing damage to cells.^23^ In this study, upon pretreatment of Ad‐PCAF RNAi, the levels of MDA and ROS were significantly reduced, while the level of SOD was increased. These results suggested that downregulation of PCAF was related to the modulation of oxidative stress pathways, PCAF downregulation increased SOD expression and corrected the imbalance between oxidation and anti‐oxidation, thereby alleviating MIRI.

As typical pro‐inflammatory cytokines, IL‐6 and TNF‐α are the main indicator of cellular inflammation.^24^ In this study, downregulation of PCAF markedly decreased the levels of IL‐6 and TNF‐α in the rat serum of MIRI and the supernatant of myocardial cell culture, revealing a possibility that downregulation of PCAF also can inhibit MIRI by reducing myocardial inflammation. In addition, the accumulation of ROS during reperfusion could cause cell apoptosis, thereby aggravating the damage of cardiomyocytes. Our results revealed that downregulation of PCAF in cells significantly reduced cardiomyocyte apoptosis and maintain the cardiac function of rat undergoing MIRI.

NF‐κB is a dimeric transcription factor composed of Rel family subunits, including 5 Rel forms, the most usual form of which is a heterodimer consisting of Rel A (p65) and p50.^25^ As a transcriptional regulatory protein, NF‐κB is widely involved in growth, differentiation, immunity, inflammation.^26^, ^27^, ^28^, ^29^ Studies have shown that NF‐κB signalling pathway plays a crucial role in the pathological process of MIRI.^30^, ^31^, ^32^ NF‐κB affects cardiomyocyte apoptosis by regulating a series of apoptosis‐related genes transcription and expression. In addition, NF‐κB is also the main target of I/R‐induced cellular oxidative stress injury.^33^, ^34^ The expression of interleukin, TNF‐α and other inflammation‐related genes were also regulated by NF‐κB.^35^, ^36^ Previous studies indicated that activation of NF‐κB promotes inflammation during myocardial ischaemia and exacerbates the heart's response to I/R injury.^37^ Inactivation of NF‐κB pathway is beneficial to alleviate I/R‐induced cardiomyocyte damage. Further mechanistic studies have found that inactivation of NF‐κB pathway has an important contribution to reducing I/R‐induced cardiomyocyte apoptosis, oxidative stress and inflammation.^38^, ^39^ In this research, we found the intervention of the expression of PCAF significantly decreased the levels of phosphorylation of NF‐κB p65 induced by I/R. Therefore, the NF‐κB pathway activator LPS was used to verify the relationship. As expected, pretreatment of LPS obviously reversed the decreased level of NF‐κB p65 phosphorylation in Ad‐PCAF RNAi treated H/R cells. The decreases of apoptosis, ROS production and pro‐inflammatory factors, such as TNF‐α and IL‐6, in the Ad‐PCAF RNAi treatment group were all reversed upon LPS pretreatment. Therefore, these results demonstrated that the protective effect of PCAF downregulation in MIRI was dependent on the inactivation of NF‐κB pathway.

## 5 CONCLUSION

Altogether, this study revealed that downregulation of PCAF could alleviate MIRI by reducing cardiomyocyte apoptosis, inflammation, and oxidative stress through regulating NF‐κB signalling pathway. Therefore, PCAF may be considered as a promising therapeutic target for MIRI.

## CONFLICT OF INTEREST

The authors declare that there is no conflict of interest.

## AUTHOR CONTRIBUTIONS

**Liqiang Qiu:** Conceptualization (equal); Data curation (equal); Formal analysis (equal); Writing‐original draft (equal). **Xiaoxiong Liu:** Conceptualization (equal); Formal analysis (equal); Methodology (equal); Writing‐original draft (equal). **Wenjing Li:** Formal analysis (equal); Methodology (equal). **Zhebo Liu:** Formal analysis (equal); Methodology (equal). **Changwu Xu:** Conceptualization (equal); Funding acquisition (equal); Project administration (equal); Writing‐review & editing (equal). **Hao Xia:** Conceptualization (equal); Funding acquisition (equal); Writing‐review & editing (equal).

## Data Availability

All data utilized in this study are included in this article, and all data supporting the findings of this study are available on reasonable request from the corresponding author (CX).

## References

[1] Hofmann U , Frantz S . Role of lymphocytes in myocardial injury, healing, and remodeling after myocardial infarction. Circ Res. 2015;116:354‐367. 25593279 10.1161/CIRCRESAHA.116.304072

[2] Jankovic N , Geelen A , Streppel MT , et al. WHO guidelines for a healthy diet and mortality from cardiovascular disease in European and American elderly: the CHANCES project. Am J Clin Nutr. 2015;102(4):745‐756. 10.3945/ajcn.114.095117 26354545 PMC4588736

[3] Yellon DM , Hausenloy DJ . Myocardial reperfusion injury. N Engl J Med. 2007;357:1121‐1135. 17855673 10.1056/NEJMra071667

[4] Chen W , Spitzl A , Mathes D , et al. Endothelial actions of ANP enhance myocardial inflammatory infiltration in the early phase after acute infarction. Circ Res. 2016;119:237‐248. 27142162 10.1161/CIRCRESAHA.115.307196

[5] Xin G , Xu‐Yong L , Shan H , et al. SH2B1 protects cardiomyocytes from ischemia/reperfusion injury via the activation of the PI3K/AKT pathway. Int Immunopharmacol. 2020;83:105910. 32222636 10.1016/j.intimp.2019.105910

[6] Qiu L , Xu C , Chen J , Li Q , Jiang H . Downregulation of the transcriptional co‐activator PCAF inhibits the proliferation and migration of vascular smooth muscle cells and attenuates NF‐κB‐mediated inflammatory responses. Biochem Biophys Res Commun. 2019;513:41‐48. 30935684 10.1016/j.bbrc.2019.03.157

[7] Zheng X , Gai X , Ding F , et al. Histone acetyltransferase PCAF up‐regulated cell apoptosis in hepatocellular carcinoma via acetylating histone H4 and inactivating AKT signaling. Mol Cancer. 2013;12:96. 23981651 10.1186/1476-4598-12-96 PMC3847488

[8] Yao W , Wang T , Xia J , Li J , Yu X , Huang F . Dietary garcinol attenuates hepatic pyruvate and triglyceride accumulation by inhibiting P300/CBP‐associated factor in mid‐to‐late pregnant rats. J Nutr. 2020;150:231‐239. 31579921 10.1093/jn/nxz238

[9] Bastiaansen AJNM , Ewing MM , de Boer HC , et al. Lysine acetyltransferase PCAF is a key regulator of arteriogenesis. Arterioscler Thromb Vasc Biol. 2013;33:1902‐1910. 23788761 10.1161/ATVBAHA.113.301579 PMC4049097

[10] Yamaguchi F , Ariga T , Yoshimura Y , Nakazawa H . Antioxidative and anti‐glycation activity of garcinol from garcinia indica fruit rind. J Agric Food Chem. 2000;48:180‐185. 10691613 10.1021/jf990845y

[11] Liu C , Ho PC‐L , Wong FC , Sethi G , Wang LZ , Goh BC . Garcinol: current status of its anti‐oxidative, anti‐inflammatory and anti‐cancer effects. Cancer Lett. 2015;362:8‐14. 25796441 10.1016/j.canlet.2015.03.019

[12] Qiu L , Xu C , Xia H , Chen J , Liu H , Jiang H . Downregulation of P300/CBP‐associated factor attenuates myocardial ischemia‐reperfusion injury via inhibiting autophagy. Int J Med Sci. 2020;17:1196‐1206. 32547315 10.7150/ijms.44604 PMC7294925

[13] Guo X , Jiang H , Yang J , et al. Radioprotective 105 kDa protein attenuates ischemia/reperfusion‐induced myocardial apoptosis and autophagy by inhibiting the activation of the TLR4/NF‐κB signaling pathway in rats. Int J Mol Med. 2016;38:885‐893. 27431018 10.3892/ijmm.2016.2686

[14] Qiu L , Xu C , Jiang H , Li W , Tong S , Xia H . Cantharidin attenuates the proliferation and migration of vascular smooth muscle cells through suppressing inflammatory response. Biol Pharm Bull. 2019;42:34‐42. 30393274 10.1248/bpb.b18-00462

[15] Shen S , He F , Cheng C , Xu B , Sheng J . Uric acid aggravates myocardial ischemia‐reperfusion injury via ROS/NLRP3 pyroptosis pathway. Biomed Pharmacother. 2021;133:110990. 33232925 10.1016/j.biopha.2020.110990

[16] Qiu L , Xu C , Jiang H , Li W , Tong S , Xia H . Cantharidin attenuates the proliferation and migration of vascular smooth muscle cells through suppressing inflammatory response. Biol Pharm Bull. 2019;42:34‐42. 30393274 10.1248/bpb.b18-00462

[17] Huang R , Shu J , Dai X , Liu Y , Yu F , Shi G . The protective effect of polyphyllin I on myocardial ischemia/reperfusion injury in rats. Ann Transl Med. 2020;8:644. 32566581 10.21037/atm-20-3371 PMC7290651

[18] Han M , Chen X‐C , Sun M‐H , et al. Overexpression of IκBα in cardiomyocytes alleviates hydrogen peroxide‐induced apoptosis and autophagy by inhibiting NF‐κB activation. Lipids Health Dis. 2020;19:150. 32580730 10.1186/s12944-020-01327-2 PMC7315514

[19] Wang S , Yang X . Eleutheroside E decreases oxidative stress and NF‐κB activation and reprograms the metabolic response against hypoxia‐reoxygenation injury in H9c2 cells. Int Immunopharmacol. 2020;84:106513. 32330867 10.1016/j.intimp.2020.106513

[20] Chen D , Lu D , Liu H , et al. Pharmacological blockade of PCAF ameliorates osteoarthritis development via dual inhibition of TNF‐α‐driven inflammation and ER stress. EBioMedicine. 2019;50:395‐407. 31735552 10.1016/j.ebiom.2019.10.054 PMC6921217

[21] Zhai M , Li B , Duan W , et al. Melatonin ameliorates myocardial ischemia reperfusion injury through SIRT3‐dependent regulation of oxidative stress and apoptosis. J Pineal Res. 2017;63:e12419. 10.1111/jpi.12419 28500761

[22] Qu D , Han J , Ren H , et al. Cardioprotective effects of astragalin against myocardial ischemia/reperfusion injury in isolated rat heart. Oxid Med Cell Longev. 2016;2016:8194690. 26788251 10.1155/2016/8194690 PMC4695676

[23] Yang B , Yan P , Yang G‐Z , Cao H‐L , Wang F , Li B . Triptolide reduces ischemia/reperfusion injury in rats and H9C2 cells via inhibition of NF‐κB, ROS and the ERK1/2 pathway. Int J Mol Med. 2018;41:3127‐3136. 29512681 10.3892/ijmm.2018.3537 PMC5881718

[24] Chen X , Li X , Zhang W , et al. Activation of AMPK inhibits inflammatory response during hypoxia and reoxygenation through modulating JNK‐mediated NF‐κB pathway. Metabolism. 2018;83:256‐270. 29526538 10.1016/j.metabol.2018.03.004 PMC5960613

[25] Wang Q , Tang XN , Yenari MA . The inflammatory response in stroke. J Neuroimmunol. 2007;184:53‐68. 17188755 10.1016/j.jneuroim.2006.11.014 PMC1868538

[26] Zarate MA , Wesolowski SR , Nguyen LM , et al. In utero inflammatory challenge induces an early activation of the hepatic innate immune response in late gestation fetal sheep. Innate Immun. 2020;26:549‐564. 32538259 10.1177/1753425920928388 PMC7556190

[27] Zhao X‐N , Bai Z‐Z , Li C‐H , Sheng C‐L , Li H‐Y . The NK‐1R antagonist aprepitant prevents LPS‐induced oxidative stress and inflammation in RAW264.7 macrophages. Drug Des Devel Ther. 2020;14:1943‐1952. 10.2147/DDDT.S244099 PMC7246327 32546961

[28] Ruhee RT , Suzuki K . The integrative role of sulforaphane in preventing inflammation, oxidative stress and fatigue: a review of a potential protective phytochemical. Antioxidants. 2020;9:521. 10.3390/antiox9060521 PMC7346151 32545803

[29] Wang Y , Li H , Xue C , et al. TRPV3 enhances skin keratinocyte proliferation through EGFR‐dependent signaling pathways. Cell Biol Toxicol. 2020;37(2):313‐330. 32535744 10.1007/s10565-020-09536-2

[30] Yuan L , Dai X , Fu H , et al. Vaspin protects rats against myocardial ischemia/reperfusion injury (MIRI) through the TLR4/NF‐κB signaling pathway. Eur J Pharmacol. 2018;835:132‐139. 30063916 10.1016/j.ejphar.2018.07.052

[31] Wang R , Wang M , Zhou J , et al. Shuxuening injection protects against myocardial ischemia‐reperfusion injury through reducing oxidative stress, inflammation and thrombosis. Ann Transl Med. 2019;7:562. 31807543 10.21037/atm.2019.09.40 PMC6861815

[32] Li D , Wang X , Huang Q , Li S , Zhou Y , Li Z . Cardioprotection of CAPE‐oNO against myocardial ischemia/reperfusion induced ROS generation via regulating the SIRT1/eNOS/NF‐κB pathway in vivo and in vitro. Redox Biol. 2018;15:62‐73. 29220696 10.1016/j.redox.2017.11.023 PMC5725281

[33] Zhao L , Zhou Z , Zhu C , Fu Z , Yu D . Luteolin alleviates myocardial ischemia reperfusion injury in rats via Siti1/NLRP3/NF‐κB pathway. Int Immunopharmacol. 2020;85:106680. 32544871 10.1016/j.intimp.2020.106680

[34] Yang X , Li C , Ng KT‐P , et al. IL‐17a exacerbates hepatic ischemia‐reperfusion injury in fatty liver by promoting neutrophil infiltration and mitochondria‐driven apoptosis. J Leukoc Biol. 2020;108:1603‐1613. 32531822 10.1002/JLB.3MA0520-716R

[35] Karunarathne WAHM , Lee KT , Choi YH , Jin C‐Y , Kim G‐Y . Anthocyanins isolated from Hibiscus syriacus L. attenuate lipopolysaccharide‐induced inflammation and endotoxic shock by inhibiting the TLR4/MD2‐mediated NF‐κB signaling pathway. Phytomedicine. 2020;76:153237. 32540784 10.1016/j.phymed.2020.153237

[36] Abdelhamid AM , Elsheakh AR , Abdelaziz RR , Suddek GM . Empagliflozin ameliorates ethanol‐induced liver injury by modulating NF‐κB/Nrf‐2/PPAR‐γ interplay in mice. Life Sci. 2020;256:117908. 32512011 10.1016/j.lfs.2020.117908

[37] Valen G , Yan ZQ , Hansson GK . Nuclear factor kappa‐B and the heart. J Am Coll Cardiol. 2001;38:307‐314. 11499717 10.1016/s0735-1097(01)01377-8

[38] Chen J , Zhang M , Zhang S , Wu J , Xue S . Rno‐microRNA‐30c‐5p promotes myocardial ischemia reperfusion injury in rats through activating NF‐κB pathway and targeting SIRT1. BMC Cardiovasc Disord. 2020;20:240. 32434515 10.1186/s12872-020-01520-2 PMC7238603

[39] Chen Y‐H , Lin H , Wang Q , Hou J‐W , Mao Z‐J , Li Y‐G . Protective role of silibinin against myocardial ischemia/reperfusion injury‐induced cardiac dysfunction. Int J Biol Sci. 2020;16:1972‐1988. 32398964 10.7150/ijbs.39259 PMC7211181

